# Treatments of pelvic girdle pain in pregnant women: adverse effects of standard treatment, acupuncture and stabilising exercises on the pregnancy, mother, delivery and the fetus/neonate

**DOI:** 10.1186/1472-6882-8-34

**Published:** 2008-06-26

**Authors:** Helen Elden, Hans-Christian Ostgaard, Monika Fagevik-Olsen, Lars Ladfors, Henrik Hagberg

**Affiliations:** 1 Perinatal Center, Department of Obstetrics and Gynecology, Institute for Clinical Sciences, Sahlgrenska Academy, Sahlgrenska University Hospital/East, Gothenburg University, Gothenburg, SE-416 85, Sweden; 2 Department of Orthopedics, Institute for Clinical Sciences, Sahlgrenska Academy, Sahlgrenska University Hospital, Goteborg University, Gothenburg, Sweden; 3 Department of Occupational Therapy and Physical Therapy, Institute of Neuroscience and Physiology, Sahlgrenska Academy, Sahlgrenska University Hospital, Gothenburg University, Gothenburg, Sweden

## Abstract

**Background:**

Previous publications indicate that acupuncture is efficient for the treatment of pelvic girdle pain, PGP, in pregnant women. However, the use of acupuncture for PGP is rare due to insufficient documentation of adverse effects of this treatment in this specific condition. The aim of the present work was to assess adverse effects of acupuncture on the pregnancy, mother, delivery and the fetus/neonate in comparison with women that received stabilising exercises as adjunct to standard treatment or standard treatment alone.

**Methods:**

In all, 386 women with PGP entered this controlled, single-blind trial. They were randomly assigned to standard treatment plus acupuncture (n = 125), standard treatment plus specific stabilising exercises (n = 131) or to standard treatment alone (n = 130) for 6 weeks. Acupuncture that may be considered strong was used and treatment was started as early as in the second trimester of pregnancy. Adverse effects were recorded during treatment and throughout the pregnancy. Influence on the fetus was measured with cardiotocography (CTG) before-during and after 43 acupuncture sessions in 43 women. A standardised computerized method to analyze the CTG reading numerically (Oxford 8000, Oxford, England) was used. After treatment, the women rated their overall experience of the treatment and listed adverse events if any in a questionnaire. Data of analgesia and oxytocin augmentation during labour, duration of labour, frequency of preterm birth, operative delivery, Apgar score, cord-blood gas/acid base balance and birth weight were also recorded.

**Results:**

There were no serious adverse events after any of the treatments. Minor adverse events were common in the acupuncture group but women rated acupuncture favourably even despite this. The computerized or visually assessed CTG analyses of antenatal recordings in connection with acupuncture were all normal.

**Conclusion:**

This study shows that acupuncture administered with a stimulation that may be considered strong led to minor adverse complaints from the mothers but had no observable severe adverse influences on the pregnancy, mother, delivery or the fetus/neonate.

## Background

Pelvic girdle pain (PGP) is common complaint in pregnant women all over the world [1]. It has a major impact on health and functioning as it decreases quality of life [2] and diminishes the capacity for standing, walking and sitting. Known risk factors for PGP are previous low back pain (LBP) and/or previous PGP and trauma [3]. PGP generally arises in relation with pregnancy, trauma and reactive arthritis. Pain is experienced between the posterior iliac crest and the gluteal fold, particularly in the vicinity of the sacroiliac joints. The pain may radiate in the posterior thigh and can occur in conjunction with/or separately in the symphysis [3]. The onset of PGP is usually by weeks 17–19 of gestation, with a peak of incidence by weeks 24–36 [4]. Postpartum follow-up studies have shown that 5–27% of the women had persisting pain 1–3 months after delivery [2,5,6] but, it has been reported that 7% have remaining pain 6 years after delivery causing severe discomfort and reduced ability to work [7].

Women affected with PGP typically adopt abnormal patterns of muscle activity, to relieve and avoid pain. The longer this pattern persists, the more pain will arise from the unphysiological burden on muscles and joints and the pain in turn will aggravate the dysfunction of muscles resulting in a vicious circle. As a consequence, the pain often becomes more severe in late pregnancy [3,8,9].

Randomized controlled trials [10-14], two systematic reviews [15,16] as well as the European guidelines for diagnosis and treatment of PGP have concluded that acupuncture is useful for treatment of PGP and low back pain, LBP during pregnancy. It has also been suggested that acupuncture is safe as a treatment option for women in early pregnancy [17]. However, the use of acupuncture in the second and third trimester of pregnancy is sparse due to several reasons. Firstly, it is considered contraindicated to puncture points in the lumbosacral region that is, somatic segments according to the innervation of the uterus for example Th 10-L2, S2–S4) [18] or to stimulate strongly in pregnancy, as it may induce preterm labor [19,20]. Secondly, although serious adverse events of acupuncture have been reported to be rare in the hands of a competent practitioner [21-26] information about long-term safety of acupuncture for PGP is lacking [11]. Most studies of acupuncture are limited only to immediate adverse events [27]. Only two studies [11,12] which are of insufficient size to draw conclusions, have reported no negative influences on birtweight and Apgar score after acupuncture for PGP and/or LBP. Thus, additional information on the safety of acupuncture during pregnancy is warranted. In addition, to our knowledge, no study has reported adverse effects of stabilising exercises on the pregnancy and the pregnancy outcome. No negative influences of the conditions themselves i.e. PGP and/or LBP on the pregnancy outcome have been found [28].

Cardiotocography (CTG) is often used in the prenatal period for detection of fetal distress[29,30]. However, it has been shown that visual assessment of CTG readings by physicians suffer from substantial intraobserver and interobserver [30,31]. Therefore, a standardised computerized method to analyze the CTG reading numerically (Oxford 8000, Oxford, England) has been developed and put into clinical practice [31].

At present, results from randomised controlled trials indicate that acupuncture and stabilising exercises as adjunct to standard treatment [16] are efficient for PGP during pregnancy. To our knowledge, no study has described the risks of adverse effects of these treatments given for PGP during the second and third trimester, on the pregnancy, mother, delivery and the fetus/neonate. In the present study we present data of adverse effects during treatment and throughout the pregnancy, influence of acupuncture on the fetus measured with cardiotocography, analgesia and oxytocin augmentation during labour, duration of labour, frequency of preterm birth and operative delivery, Apgar score, cord-blood gas/acid base balance and birth weight of the neonates.

## Methods

This paper is an additional report of further results from a large study earlier presented in 2005 [13]. The delay in time for publication of the specific data in this paper was mainly caused by the time consuming first publishing progress. Women were recruited to the study from 27 antenatal clinics in the Sahlgrenska University hospital reference area. The study was approved by the local Ethics Committee of the University in Gothenburg and it was performed between August 2000 and May 2002 in Gothenburg, Sweden. Women were eligible for the study if they were healthy and at 12–31 completed gestational weeks with singleton fetuses; spoke Swedish fluently, gave their informed consent and were diagnosed with PGP according to Ostgaards criteria [4] i.e. time- and weight bearing related pain, pain-free intervals with sudden pain attacks, pain when turning in bed, a free range of motion in the hips and spine, no nerve root syndrome and a positive posterior pelvic pain provocation test. Women were ineligible for the study if they had other pain conditions; history of orthopedic disease or surgery in the spine or pelvis, systemic disorders, coagulation disturbances or increased risk of infection. The study comprised a one-week baseline period, 6 weeks of treatment; follow up within one week after the last treatment. After informed consent from the women and performance of the baseline assessment, an independent examiner randomised the patients individually to one of the treatment groups. The full examination programme and randomisation procedure have been described previously [13]. Mean maternal age in the standard group was 30.4 (SD 4.7), 30.5 (4.4) in the acupuncture group and 29.8 (4.2) in the stabilising group. Thirty-three women (25.6%), 34 (27.4%) and 36 (27.7%) respectively were primipara.

### Standard treatment

Standard treatment consisted of basic information about PGP and anatomy of the back and pelvis. The purpose of this information was to reduce fear, and to encourage patients to become active in their own treatment. The patients got a pelvic belt and a traditional home training programme including easy performed exercises for global muscles, with aim to increase strength in the abdominal, back, gluteal and shoulder muscles. Patients were told that they were free to call the physiotherapist at any time if they had questions about the home exercises or wanted more help i.e. needed crutches.

### Acupuncture

The same treatment as in the standard group, but in addition acupuncture. The aim with acupuncture was to establish control of PGP to prevent dysfunction of muscles of the spine and pelvis. The aim with the stimulation was to activate both the segmental pain inhibitory system, involving the so-called gate control mechanism [32] and the central pain inhibitory system, involving secretion of endogenous opioids [33]. The number and selection of acupuncture points was based on clinical experience and expert knowledge of acupuncture in pregnant women with PGP. Ten classical acupuncture points were selected individually in the same segments as the location of PGP after diagnostic palpation to identify sensitive spots (Table 1). Two acupuncture points on the medial side of the leg and foot were selected in the same segment as the PGP and extra-segmental points to the lumbosacral area were used to strengthen and lengthen the effect of the central control systems.

**Table 1:** Acupuncture points, their innervations, and their anatomical positions

**Points**	**Segmental innervation**	**Tissue localisation**
GV 20	N trigeminus (V), occipitalis minor (C2), occipitalis major (C2-3)	Aponeurosis epicrani tissue
LI 4 bilateral	N ulnaris medianus (C8, Th 1)	M interosseus.dorsalis I lumbricalis II, adduktor pollicis
BL 26 bilateral	N thoracodorsalis (C6-8), toracicus (Th 9–12), lumbalis (L1-3)	N. thoracolumbalis, m erector spinae
BL 32 bilateral	N thoracodorsalis (C6-8), toracicus (Th 9–12), lumbalis (L1-3)	Fascia thoracolumbalis, m erector spinae
BL 33 bilateral	N. thoracodorsalis (C6-8) toracicus (Th 9–12), lumbalis (L1-3)	Fascia thoracolumbalis, m erector spinae
BL 54 bilateral	N gluteus inferior (L5, S1-2)	M gluteus maximus
KI 11 bilateral	N thoracicus (Th 6–12), subcostalis	Vagina m. recti abdominis
BL 60 bilateral	N. suralis (S2)	Fibrotic tissue
EX 21 bilateral	N lumbalis, sacralis (L4-5, S1-2)	Fascia thoracolumbalis, m. erector spinae
GB 30 bilateral	N gluteus inferior (L5, S1), obturatorius internus (L4-5, S1)	M gluteus maximus, gemellus superior, piriformis
SP 12 bilateral	N. thoracicus, lumbalis (Th 7–12, L1)	Aponeurosis mm. obliquus externus, abdominus internus
ST 36 bilateral	N. peroneus profundus (L4-5)	M tibialis anterior

LI, large intestine channel; BL, bladder channel; KI, kidney channel; GB, gall bladder channel; ST, stomach channel; GV, governor vessel channel

The needles (Hegu; Hegu AB, Landsbro, Sweden) were disposable and made of stainless steel (∅ 0.30) and they were inserted intramuscularly to a depth of 15–70 mm. Needles were manipulated manually to evoke needle sensation (De Qi), a characteristic feeling of tension, local pain, soreness or numbness, and often a radiating feeling from the point of insertion. The needles were manually stimulated every ten minutes and left in situ for 30 minutes. Treatment was given twice a week.

### Stabilising exercises

The same treatment as in the standard group but, in addition, stabilising exercises and massage [34]. The aim was to retain motor control by retaining a co-contraction pattern of the deep trunk muscles [35], to correct posture and avoid negative stress to pelvic structures in daily life [34,36,37]. The exercises which caused low load on the pelvic structures were given according to Richardson and Jull [34] but modified because of the pregnancy. All exercises were carefully instructed individually, but performed as home-exercises on a daily basis. Exercises started with isolated low force co-contractions in M. Transversus Abdominis and Mm Multifidii in static postures. These exercises were, followed by increasingly more difficult challenges as the patient's skills improved. The aim with the massage was to increase blood-flow in the gluteal muscles and hip extensors. It was given once or a few times by the physiotherapist, the patient and partner was thereafter instructed how to perform the massage at home.

### Recording of adverse events

The women receiving acupuncture and stabilising exercises were asked whether they had identified a side effect at each visit, although they were not presented with a list of possible side effects. However, before the first treatment the women in the acupuncture group were informed of possible side effects connected to acupuncture. I.e. they were informed that bleeding or bruising, pain on needling, and aggravation of symptoms occur in some patients and that they could experience drowsiness, especially after the first treatment. Patients were also advised to avoid strenuous physical activity the same day as the treatment. Immediate adverse events connected to acupuncture as hypotension/fainting and/or nausea were recorded. Fetal heart rate, maternal heart rate and blood pressure were monitored before and after all acupuncture sessions to assess the cardiovascular effect of acupuncture and influence on the fetal heart rate was measured with CTG in 43 women. CTG was recorded prior, during and after 43 treatment (30 minutes each time or until the trace was approved as being normal). The CTG traces were assessed both by the computer (Oxford 8000 computerised CTG analyser, Oxford Sonicaid Ltd, Oxford, England) and visually by an experienced obstetrician [31].

The women in the stabilising exercise group were informed that PGP could aggravate if they overloaded their pelvis and they were instructed to lower the intensity of the exercises if this happened.

At follow-up visit to the physiotherapist, (who was not informed of treatment allocation), all women filled out a questionnaire in which they answered a question if they had experienced harm of the treatment they had received (no or yes). If they marked yes, they were asked to list disadvantages and/or adverse events that they had experienced. The women were also asked to indicate their overall experience of the treatments. The options were: no help, some help, good help and very good help.

To assess influence of treatments on the pregnancy, delivery and condition of the child at birth, we recorded antenatal, intrapartum, neonatal and infant data that are normally registered in the Medical Birth Register (MBR) in Sweden. Assessment of the condition of the child at birth was based on weight, cord-artery and vein acid-base status (pH, and base deficit, mmol/L) and Apgar score. In Sweden preterm birth is defined as < 37 weeks gestation, metabolic acidosis is defined as cord artery pH ≤ 7.05 and BDef ≥ -12 and neonatal death is defined as death of a live born infant within 28 days of birth.

Data analyses were performed by a statistician who was blinded to group and treatment. Continuous data were tested for significance with Kruskal-Wallis test. If a significant difference was found between any of the three groups, Mann Whitney U test was used to compare one group against one another. Dichotomous data were tested for significance with Fischer's exact test. Adjustments (multiplication by three) of the p-values due to multiple comparisons were performed by Bonferroni's method when appropriate. An adjusted p-value < 0.05 was considered statistically significant. Calculation of statistical tests and descriptive evaluations were carried out using SAS software package, version V8.

## Results

**Figure 1:**
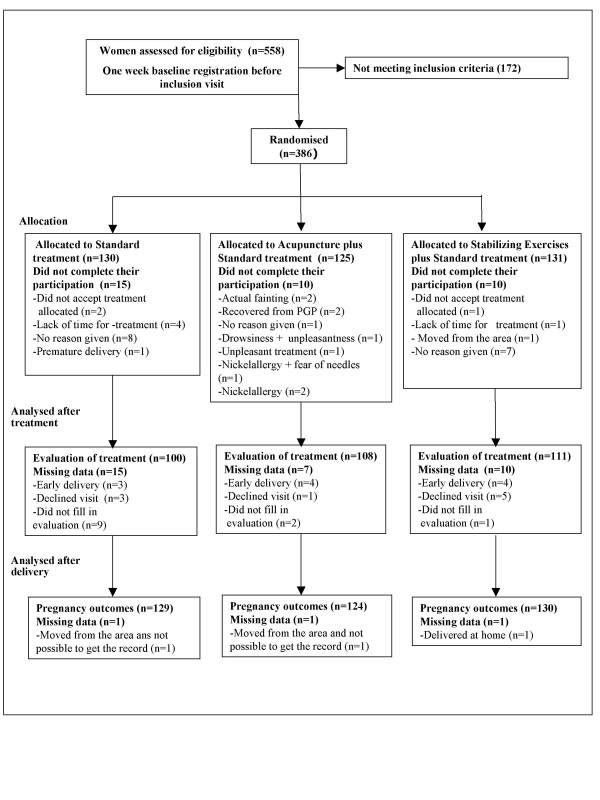
Flow-chart of the study.

A total of 386 subjects were randomised (Fig. 1), 35 women did not complete the treatment sessions. Even though pain relieving effect was only evaluated in cases completing treatment, side effects were recorded in all the cases randomised except for one woman that delivered at home and two women moving out of town (Fig. 1). No differences between the groups regarding baseline characteristics were found (Table 2). There were no differences between the women who withdrew during the trial and those who completed therapy, with respect to adverse events during pregnancy, pregnancy complications or pregnancy outcomes.

**Table 2:** Outcome variables of deliveries among 383 women with PGP during pregnancy

**Outcomes**			
	**Standard group (n = 129)**	**Acupuncture group (n = 124)**	**Stabilising exercise group (n = 130)**

Mean gestation week at delivery±	39.5 (1.6)	39.2 (1.7)	39.7 (1.6)
Gestation week at delivery (min-max)	27–43	34–43	34–43
Induction of labour§	7 (5.4)	15 (12)	12 (9.2)
Spontaneous contractions§	113 (87.6)	103 (83.1)	112 (86.1)
Established contractions to delivery, hours±	7.7 (8)	6.4 (5)	6.2 (4)
Use of oxytocin§	33(25.6)	37 (29.8)	31 (23.80)
Spontaneous delivery§	107 (82.9)	93 (75)	106 (81.5)
Preterm delivery (<37 gestation weeks) §	7 (5.4)	5 (4.0)	7 (5.4)
Duration of second stage of labour in minutes±	31 (25)	36 (35)	32 (34)
Vaginal operative delivery§	3 (2.3)	5 (4.0)	3 (2.3)
Caesarean section§	16 (12.4)	19 (15.3)	18 (13.8)
Episiotomy§	8 (6.2)	9 (7.31)	6 (4.6)
Third or fourth degree tears§	5 (3.9)	4 (3.2)	3 (2.3)

§Numbers reported with percentage of group.

±Mean scores are reported with standard deviations.

No significant differences were recorded between groups.

### CTG readings

The computerized or visually assessed CTG analyses of antenatal recordings in connection with acupuncture were all normal (Table 3).

**Table 3:** Descriptive data in 43 cases undergoing CTG recordings

**Outcomes**	
Mean gestational week±	33+3 (1+2)
Range gestational week±	32–37
Mean maternal age, years±	31.5 (4)
First pregnancy§	11 (25.8)
Blood pressure before acupuncture±	117/67 (11/9)
Blood pressure after acupuncture±	117/69 (11/9)
Maternal heart rate before acupuncture±	80 (8)
Maternal heart rate after acupuncture±	79 (8)
Fetal heart rate before acupuncture±	137 (9)
Fetal heart rate after acupuncture±	136 (9)

§Numbers reported with percentage of CTG group.

±Mean scores are reported with standard deviations.

### Serious and minor adverse events

There were no serious adverse events associated with either of the treatments. Minor adverse events were most common in the group receiving acupuncture (Table 4).

**Table 4:** Details of minor adverse events associated with treatments reported during the treatment period by the patients in the study

**Adverse events**			
	**Standard Group (n = 130)**	**Acupuncture Group (n = 125)**	**Stabilising exercise group (n = 131)**

Number of adverse events	8 in 8 women	64 in 43 women	22 in 22 women
Painful treatment	-	23 experienced pain from needles	-
Worsening of PGP	During and after exercises (n = 2)	During the same day that they had received treatment (n = 15)	During the exercises (n = 2)
Back pain	-	-	Thoracic pain (n = 1) LBP in the beginning of the treatment (n = 17)
Treatment discomfort-table	Rubbing from pelvic belt (n = 5)	Unpleasant treatment (n = 5)	-
Drowsiness after treatment	-	(n = 12)	-
Psychological and emotional reactions	-	Intense emotional release, (feeling energised) (n = 1)	-
Miscellaneous symptoms	Uterine contractions during exercise (n = 1)	Headache plus severe drowsiness the same day as the treatment and the day after (n = 1); headache the day of the treatment (n = 1); rash developed on the place of the needles a few days after treatment (n = 2); severe nausea with feeling faint, sweating and dizziness (n = 4).	Premature bleeding (n = 1); premature contractions (n = 1)

### Women's opinions of the treatments

The acupuncture group and the stabilising exercise group were significantly more likely to be "very satisfied" with their treatment than the standard treatment group (Table 5).

**Table 5:** Participants opinions of the treatment

**Outcomes**					
	**Standard group (n = 100)**	**Acupuncture group (n = 108)**	**Stabilizing exercise group (n = 111)**	**P-value**	

No help of treatment	25 (25)	4 (4)	2 (2)	S-ACU	< 0.001
				SE-S	< 0.001
				ACU-SE	NS
Some help of treatment	53 (53)	21 (19)	28 (25)	S-ACU	< 0.001
				SE-S	< 0.001
				ACU-SE	NS
Good help of treatment	14 (14)	37 (34)	38 (34)	S-ACU	< 0.001
				SE-S	< 0.001
				ACU-SE	NS
Very good help of treatment	8 (8)	46 (43)	43 (39)	S-ACU	< 0.001
				SE-S	< 0.001
				ACU-SE	NS
Experienced harm of treatment	51 (51)	43 (40)	22 (20)	S-ACU	0.674
				S-SE	<0.001
				ACU-SE	<0.001
Painful treatment	1 (1)	27 (24)	3 (3)	S-ACU	<0.001
				S-SE	NS
				ACU-SE	<0.001
Treatment not enough	13 (13)	0	3 (2)	S-ACU	<0.001
				S-SE	0.014
				ACU-SE	NS
Difficult to exercise at home	24 (24)	0	5 (4)	S-ACU	<0.001
				S-SE	<0.001
				ACU-SE	NS
Syncope	0	9 (8)	0	All groups	NS
Drowsiness after treatment	0	9 (8)	0	S-ACU	0.011
				ACU-SE	0.004
Did not relieve pain	3 (3)	2 (2)	5 (4)	All groups	NS
Pelvic belt uncomfortable	5 (5)	0	0	S-ACU	NS
				S-SE	NS
Treatment unpleasant	0	7 (6)	0	S-ACU	0.020
				ACU-SE	0.044
Time consuming	7 (7)	5 (4)	4 (3)	S-ACU	NS
				S-SE	NS
				ACU-SE	NS
Would choose the same treatment again	69 (69)	98 (93)	106 (95)	S-ACU	<0.001
				S-SE	<0.001
				ACU-SE	NS

S, standard treatment; ACU, acupuncture plus standard treatment; SE, stabilizing exercises plus standard treatment

Numbers reported with percentage of group

§Numbers reported with percentage of group.

±Mean scores are reported with standard deviations.

Data were tested for significance with Fischer's exact test. Adjustments (multiplication by three) of the p-values due to multiple comparisons were performed by Bonferroni's method. NS = Not significant.

### Pregnancy outcomes

No negative influence on the pregnancy outcomes were found after either of the treatments (Table 2). Acupuncture did not lead to an increased rate of preterm delivery, five women (4%) in the acupuncture group (two in week 34 and 3 in week36) delivered preterm. Frequency of pre-term delivery was similar in the two other treatment groups, seven women (5.4%) in the standard group (two in week 34 and 35 and three in week 36 respectively) and seven women (5%) in the stabilising exercise group (one in week 34, two in week 35 and four in week 36 respectively) delivered preterm. There were no differences between groups with regard to pregnancy complications or adverse clinical conditions (Table 6).

**Table 6:** Averse events on the pregnancy reported in participants records

**Clinical conditions**			
	**Standard group (n = 130)**	**Acupuncture group (n = 125)**	**Stabilising exercise group (n = 131)**

Preeclampsi	5	2	2
Anaemia	1	-	-
Antepartum haemorrhage	-	-	1
Ablatio placenta	-	-	1
Abdominal pain	2	-	-
Diabetes White A	1	3	2
Hydronefrosis	1	-	-
Carpal tunnel syndrome	-	2	1
Gastritis	1	-	-
Premature contractions	1	-	1
Rhesus incompatibility gestational week 35	1	-	-

Values are n.

### Analgesia during labour

Table 7 shows that the usage of analgesia was similar in the three treatment groups.

**Table 7:** Analgesia used during 383 deliveries among women with PGP during pregnancy

**Analgesia**			
	**Standard Group (n = 129)**	**Acupuncture Group (n = 124)**	**Stabilising exercise group (n = 130)**

Nitrous oxide/oxygen	101 (78.3)	87 (70.2)	100 (76.9)
Tub bath	21 (16.3)	16 (12.9)	26 (20.0)
TENS	5 (3.9)	4 (3.2)	5 (3.8)
Acupuncture	20 (15.5)	18 (14.5)	26 (20.0)
IC sterile water	7 (5.4)	2 (1.6)	7 (5.4)
Infiltration of local anaesthetic	34 (26.4)	39 (31.4)	34 (26.1)
Pethidine	0	0	0
Pudendal nerve blockade	4 (3.1)	6 (4.8)	3 (2.3)
Paracervical blockade	1 (0.8)	5 (4.0)	2 (1.5)
Epidural analgesia	30 (23.3)	32 (25.8)	31 (23.8)
Spinal analgesia	16 (12.4)	13 (10.5)	11 (8.5)
General Anaesthesia	4 (3.1)	6 (4.8)	8 (6.2)

Numbers reported with percentage of group.

± Mean scores are reported with standard deviations.

Data were tested for significance with Fischer's exact test. Adjustments (multiplication by three) of the p-values due to multiple comparisons were performed by Bonferroni's method when appropriate. There were no significant differences between groups.

### Neonatal outcome

Table 8 shows that there were no differences detected between the study groups in neonatal outcomes. Acupuncture did not lead to credible negative influences on the fetuses or neonates. There was one perinatal death related to meconium aspiration combined with infection and pneumothorax reported in the stabilising exercise group. There were four neonates in the acupuncture group, one neonate in the standard group and two neonates in the stabilising group with metabolic acidosis.

**Table 8:** Mean outcome variables [with standard deviations] of 379 newborns

**Characteristics of newborns**			
	**Standard Group (n = 129)**	**Acupuncture Group (n = 124)**	**Stabilising exercise group (n = 130)**

Weight (g) ±	3667 (548)	3576 (521)	3684 (523)
Sex (girls)§	52 (40.3)	74 (59.7)*	62 (47.7)
Sex (boys)§	77 (59.7)	50(40.3)	68 (52.3)
Apgar score ≤ 7 at 5 min§	1 (0.8)	1 (0.8)	2 (1.5)
Admission to special care baby unit§	6 (4.6)	6 (5)	9 (7)
Perinatal mortality§	0	0	1 (1)
pH±	7.23 (0.1)	7.23 (0.01)	7.22 (0.1)
PCO2±	6.63 (2.6)	6.23 (2.1)	6.41 (2.5)
PO2±	3.26 (2.5)	4.02 (2.7)	3.68 (2.9)
BEt±	-5.51 (3.2)	-5.15 (7.4)	-5.38 (4.9)
*Number with no sample§	38 (30)	40 (31)	38 (29)
pH±	7.31 (0.1)	7.26 (0.4)	7.32 (0.1)
PCO2±	5.28 (1.1)	5.13 (1.1)	5.18 (1.3)
PO2±	4.15 (1.7)	4.30 (2.1)	4.41 (1.8)
BEt±	-6.26 (8.6)	-4.28 (8.5)	-4.91 (9.8)
*Number with no sample§	39 (30)	33 (32)	31 (24)

§Numbers reported with percentage of group

± Mean scores are reported with standard deviations.

*Cord-blood acid base analysis not done in every case.

Continuous data were tested for significance with Kruskal-Wallis test. If a significant difference was found between any of the three groups, Mann Whitney U test was used to compare one group against one another. Dichotomous data were tested for significance with Fischer's exact test. Adjustments (multiplication by three) of the p-values due to multiple comparisons were performed by Bonferroni's method when appropriate.

** Indicate statistical difference between Standard treatment and Acupuncture, P = 0.01.

## Discussion

The main result of this study is acupuncture, which may be considered strong, given during the second and third trimester of pregnancy, does not lead to serious adverse events in women during pregnancy and delivery or in fetuses/neonates. As, expected, no serious adverse events were found after standard treatment and stabilising exercises. All but one of the women in each group that withdraw from treatment are included in the thorough follow-up which guarantees that there were no long-term adverse events in these women.

### Standard treatment

We are not surprised that adverse events were rare in the standard group as the prescribed exercises were of low-force. Experience of slight rubbing connected with use of the semi plastic belt have also been reported by others [38-42].

### Acupuncture

Our result shows that although minor adverse events were more frequent in the acupuncture group than in the other study groups, they were sparse. Only 64 minor adverse events (5%) out of 1414 acupuncture treatments were reported. These minor adverse events were reported by 43 women showing that some women were more susceptible than others. Also, six out of ten of women who withdrew from acupuncture treatment reported one of these adverse events as reason for not completing their participation. The most frequent adverse events were pain at the site of needling, drowsiness after treatment and headache, which is in line with a postal survey of 9408 prospectively identified acupuncture patients [25]. Our data of no serious adverse events in pregnant women during the treatment course of acupuncture are also in agreement with previous studies of PGP and/or LBP during pregnancy [10-12,14].

We believe that experience of needle pain (n = 27) must be considered unavoidable when treatment involves needling. In addition, the pain may have resulted from the achievement of de qi, a feeling thought to be essential for a successful treatment. Nevertheless, in spite of the minor adverse events, the women rated acupuncture favourably even though this and a majority of them were willing to use the same treatment in the future if needed, suggesting that the pain of the treatment did not negatively impact their overall experience of the treatment.

We think that fear of immediate and long-term serious adverse events of acupuncture in pregnant women have resulted in both a restricted use of acupuncture and a delayed start of this treatment in pregnant women. To reduce development of abnormal patterns of muscle activity [3] we decided to begin treatments as soon as possible after the first symptoms of PGP. As a consequence, 62 women (50%) in the acupuncture group started treatment as early as in gestational week 14 to 25.

In addition, an adequate dose of acupuncture probably is essential for the outcome of treatment and, as no scientific evidence of harm of acupuncture during pregnancy exists we decided not to reduce the stimulation because of the pregnancy. Therefore we used acupuncture points and the same stimulation that is normally used in a non pregnant population. Fortunately, this strategy resulted in significant pain relieving effects [13]. It is not easy to find safety data of acupuncture given to women in the second and third trimester of pregnancy because these women are seldom offered acupuncture due to the fact that pregnancy sometimes is regarded as a relative contraindication to acupuncture (because of an old Chinese proposal of an increased risk of uterine contractions leading to abortion or pre-term delivery. There are only four other studies published (171 women in total) of acupuncture for PGP and/or LBP [10-12,14]. Two of them (n = 71) registered Apgar score and infant weight. However these studies used weaker stimulation than in our study. Kvorning et al. [11] started treatment with only two segmental points, stimulated to achieve de-qi twice during the session which only lasted for about three minutes. The needles were than withdrawn and the patient was allowed to rest for ten minutes [11]. Guerreiro da Silva et al. [12] used 8 to 12 sessions with 12 needles for 25 minutes. In addition, Smith et al. [17] who treated 583 pregnant women with acupuncture for hyperemesis during the first trimester [17] reported no influences on the pregnancy outcome. But as in our study the sample size in that study was only able to detect large differences in pregnancy outcomes. These authors declared that a sample size of 19,476 women would have been required to detect an increased from 6 to 7% in spontaneous abortions.

### Physiotherapy/Stabilising exercises

There were 22 minor adverse events reported by 22 women in the stabilising exercise group. The most frequent adverse event was pain in the lower back at the beginning of the treatment period (n = 17). Our results of no serious adverse events of physiotherapy are in agreement with earlier research [10,11,41-51]. In addition, 115/131 (88%) women in our study started treatment after gestational week 18 and a prospective epidemiological study of 92 671 pregnant women have reported no association between exercise performed after 18 weeks of gestation and risk of miscarriage [52].

### CTG readings

Our results of no signs of fetal distress in connection with acupuncture is supported by Neri et al. [53] who found no signs of fetal distress or changes in short-or long-term variability on computerized CTG before, during and after minimal and true acupuncture plus moxibustion for breech presentation in 15 pregnant women. In addition, two prior studies of acupuncture have showed beneficial effect on the blood flow velocity ratios in the uterine arteries, which may be beneficial also for the fetus. One study was performed in infertile women and the other in women with uncomplicated pregnancies. The first study [54] showed a significant decrease of the mean pulsatility index, PI, in the uterine arteries after eight electro-acupuncture treatments reduced resistance in the placental vascular bed. The other study [55] showed that insertion of acupuncture needles at LI-4 plus at SP-6 lead to a significantly decrease of the uterine S/D ratios from 2.45 ± 0.3 to 2.22 ± 0.2 after 30 minutes indicating a positive influence on uterine artery waveforms. Possible mechanisms behind these results are a decreased tonic activity in the sympathetic vasoconstrictor fibres to the uterus and involvement of central mechanisms with general inhibition of the sympathetic outflow, in accordance with previously observed acupuncture effects [54]. However, the mechanism which leads to an alteration of blood flow velocity and vascular tonus after acupuncture treatments is not well understood and to our knowledge the clinical benefit of a moderate decrease of the uterine S/D ratios from 2.45 ± 0.3 to 2.22 ± 0.2 in uncomplicated pregnancies is questionable.

### Use of pain relief in labour, parturition and neonatal outcome

As described in the method section, we used the same antenatal, intrapartum, neonatal and infant data that has been complied on all births in Sweden by the Swedish MBR since 1973. This register offers a unique possibility to obtain reliable prevalence figures regarding obstetric and neonatal outcome. Unfortunately this register has no data of frequency of acupuncture during the antenatal period. If that had been the case it would have been easy to collect data on safety from all pregnant women in Sweden that had got acupuncture for PGP and/or LBP.

The three different treatments were not found to affect the use of pain relief in labour, parturition or neonatal outcome. Effects on labour or parturition or cord blood acid base/gas balance after acupuncture treatment for PGP have not been reported earlier. Analysis showed that four neonates in the acupuncture group and two neonates in the stabilising exercise group had metabolic acidosis in the arterial cord blood. However, only one of the neonates in the standard group, one neonate in the acupuncture group and two of the neonates in the stabilising exercise group had Apgar score ≤ 7 at 5 minutes indicating metabolic acidosis of any importance. One limitation of the study is that cord blood was analysed in only 70% of the neonates. We assume that the generalizability of our results is not biased by selection effects as the frequency of cord blood analysis did not differ from all deliveries at Sahlgrenska University hospital during the same period.

The frequency of induction of delivery, oxytocin augmentation, preterm delivery, and postmaternity were similar in the treatment groups. Nor did acupuncture lead to observable negative influences on the neonates in our study, yet, the study was not powered to fully exclude such influences. In Sweden, the frequency of neonatal death is 0.3% and the frequency of preterm delivery is 5.6%, thus a very large number of patients would have been required to fully investigate these issues.

Our results are in line with a previous published review [56] of acupuncture for labour pain, which showed no negative effects on the delivery or the newborns. However, the women in our study received treatments that was considered "strong" (17 needles during 12 sessions), which can not be comparable to acupuncture given only during labour. A secondary finding in our study was that the distribution between male and female fetuses. One previous epidemiological study has reported that male sex of the fetus was related to back pain symptoms during pregnancy [57] However, this was not seen in our study, of the 383 neonates 48.7% were female and 51.3% were male as expected.

### Clinical implications

When trying to determine the clinical implications of the present study we have to consider that

1. The data emerged from a relatively large randomised trial, although we are aware that an efficacy randomised controlled trial is not an appropriate design to provide evidence of safety. Studies of more than 97 000 participants' have been used in the past [26].

2. A substantial number of women started treatment already during the second trimester of pregnancy.

3. The acupuncture may be considered to be strong compared to earlier studies. We used 17 needles, 10 of them were located in the lumbosacral region (i.e. somatic segments according to the innervation of the uterus that is, Th 12-L2, S2–S3) [18]. They were stimulated to de qi 3 times during 30 minutes and, acupuncture points were used that have been stated by others [19,20] to be avoided during pregnancy (LI 4, BL 31 and 33).

4. The investigation of the complications during pregnancy, pregnancy outcomes and the neonates were quite extensive compared to earlier published studies.

5. But the study was of insufficient size to exclude negative effects on preterm delivery, perinatal morbidity and mortality as well as on CTG.

## Conclusion

In conclusion, this is the first large clinical trial of standard treatment, stabilising exercises and acupuncture administered with stimulation that may be considered strong for PGP to report on effects on CTG, labour, parturition and cord blood gas/acid base status. The study result adds support to the view that acupuncture even with stimulation that may be considered as strong is not accompanied by any severe adverse influences on the pregnant women or the fetus/neonate. The data have important clinical implications in eliminating fear of serious adverse effects when treating women with acupuncture during the second and third trimester of pregnancy. Even if more studies are required, the present study provides the most comprehensive data reported to date.

## Abbreviations

ACU: acupuncture plus standard treatment; BL: bladder channel; CTG: cardiotocography: GB: gall bladder channel; KI: kidney channel; LBP: Low back pain; LI: large intestine channel; MBR: medical birth register; PGP: pelvic girdle pain; S: standard treatment; SE: stabilizing exercises plus standard treatment; ST: stomach channel; GV: governor vessel channel.

## Competing interests

The authors declare that they have no competing interest.

## Authors' contributions

All investigators have access to all data in the study. HE initiated and coordinated the study, did most of the data collection, contributed to study design and interpretation of the results. LL advised in data collection and assisted in study design and in the statistical analysis of the data. MF-O assisted in study design and gathered data and assisted in the statistical analysis of the data. HC-O guided the scientific process, assisted in study design, provided advice on the epidemiology of pelvic girdle pain and contributed to funding of the project. HH guided the scientific process, assisted in study design and provided funding. All investigators contributed to data interpretation and preparation of the manuscript. HC-O and HH are guarantors for the study.

## Pre-publication history

The pre-publication history for this paper can be accessed here:


